# HIV infections and HIV testing during pregnancy, Germany, 1993 to 2016

**DOI:** 10.2807/1560-7917.ES.2019.24.48.1900078

**Published:** 2019-11-28

**Authors:** Ulrich Marcus

**Affiliations:** 1 Robert Koch-Institut, Berlin, Germany

**Keywords:** HIV, mother-to-child transmission, Germany, HIV pregnancy screening

## Abstract

**Background:**

Elimination of mother-to-child transmission (MTCT) of HIV by 2020 is a goal of the World Health Organization (WHO) action plan for the European Region. However, data to monitor progress towards MTCT elimination are not readily available in Germany.

**Aim:**

We aimed to estimate the number of pregnant women with HIV and MTCT rates in Germany.

**Methods:**

We triangulated retrospectively obtained data from: (i) healthcare reimbursement for HIV screening tests, (ii) a statutory health insurance subsample of prevalent and incident HIV diagnoses among pregnant women, (iii) a mathematical model of the German HIV epidemic with number, region of origin and risk factors for women of childbearing age, and (iv) the statutory anonymous HIV registry on children infected through HIV MTCT.

**Results:**

The number of women aged 15–49 years with HIV increased from ca 6,000 in 1993 to ca 11,000 in 2016. Risk of injecting drug use (IDU) declined from 65% in 1993 to 16% in 2016. The annual proportion of women living with HIV giving live birth increased from a mean of 1.9% during 1993 to 1998 to 4.9% in 2011 to 2015. HIV screening rates during pregnancy increased from ca 50% in 2001 to ca 90% in 2016. The HIV MTCT rate dropped from 6.8% in 2001 to 1.1% in 2016.

**Conclusions:**

The population of women living with HIV in Germany shifted from predominantly IDU-associated infections to predominantly sexually acquired infections, while fertility rates more than doubled. MTCT rates dropped, mainly because of improved detection and management of HIV in pregnancy.

## Introduction

In 2016, the World Health Organization Regional Office for Europe (WHO/Europe) published an ‘Action plan for the health sector response to HIV in the WHO European Region’ [1]. It announced the WHO target to eliminate mother-to-child transmission (MTCT) of HIV in the European region by 2020 .

The elimination targets specific to the prevention of MTCT were to: (i) reduce MTCT to < 2% in non-breastfeeding populations and (ii) reduce the rate of HIV and congenital syphilis in infants. This should be achieved by setting national targets, expanding coverage with antenatal care and testing (including in key populations), providing lifelong antiretroviral treatment (ART) for women during pregnancy and after delivery, and ensuring early diagnosis of infants and immediate treatment for all infants diagnosed with HIV.

Two indicators were proposed to measure progress towards these targets: (i) HIV MTCT rate, i.e. the percentage of infants born to HIV-positive women in the past 12 months who become infected with HIV and (ii) HIV MTCT case rate, i.e. the number of new congenital HIV MTCT cases per 100,000 live births.

Effective measures to prevent or reduce MTCT have been established: (i) Caesarean section to reduce exposure to maternal blood during delivery [2], (ii) antiretroviral treatment during pregnancy with the goal to have an undetectable viral load at delivery [3,4]; (iii) postpartum post-exposure prophylaxis (PEP) for the newborn with antiretroviral drugs [5], and (iv) formula feeding instead of breastfeeding to avoid HIV transmission via breastmilk [6]. Meanwhile it has been shown that Caesarean section has no additional benefit if maternal viral load is undetectable at delivery, and it is debated whether postpartum PEP for the newborn is still required if maternal viral load was undetectable during the last weeks of pregnancy [7]. The need for formula feeding is also under discussion if the mother is effectively treated for HIV [8].

In Germany, prenatal care is guided by maternity care guidelines that include recommendations on testing and other preventive activities for prenatal care. Pregnant women receive a ‘maternity passport’ in which the attending physician documents tests and findings relevant for prenatal care. Table 1 shows the history of inclusion of HIV screening into these prenatal care guidelines. Recommendations on HIV testing and on how to manage HIV infection during pregnancy and delivery were first introduced in Germany during the 1990s in specific clinical HIV pregnancy guidelines. The first general maternity care guidelines that included recommendations on HIV were only published in 2003 [9].

**Table 1:** Evolution of prenatal care guidelines with respect to HIV^a^, Germany, 1985–2015

Guideline version	HIV-related recommendations
1985 Prenatal care guidelines	HIV not mentioned. Generic recommendations regarding serological testing for infections and appropriate treatment and care for infections.
1990s HIV and pregnancy guidelines	Specialist guidelines for HIV and pregnancy: recommendations on HIV testing and on how to manage HIV infection during pregnancy and delivery (regularly updated since then). While these guidelines are usually comprehensive and up to date, they are not well known among general gynaecologists.
2003 Prenatal care guidelines	First version of general prenatal care guidelines including HIV screening recommendations: ‘voluntary serological testing for HIV antibodies after medical counselling about HIV and MTCT transmission risks, if appropriate’. HIV screening was recommended at the first antenatal screening visit within the first 12 weeks of pregnancy. There was no space in the ‘maternity passport‘ to document HIV testing (in contrast to all other recommended screening tests).
2007 Prenatal care guidelines	Inclusion of an explicit statement that an HIV screening test should be recommended to every pregnant woman.
2015 Prenatal care guidelines	Introduction of a tick box in the ‘maternity passport‘ to indicate whether an HIV screening test had been performed.

^a^ The German prenatal care guidelines are the essential resource for the delivery of prenatal care in Germany.

The strategic approach for preventing HIV MTCT in Germany since the mid-1990s has included universal voluntary HIV testing during pregnancy, offering treatment to all pregnant women found to be infected, including postnatal infant prophylaxis if necessary. Medical guidelines on HIV treatment and MTCT prophylaxis during pregnancy, delivery and in the immediate postnatal period have been updated regularly [10].

Unfortunately, in Germany as in many other countries, it is difficult to monitor progress in the prevention of HIV MTCT because of a lack of appropriate data sources for some key data. In Germany, there are no readily available national-level data on the number of: (i) pregnant women and women delivering a baby who have been tested for HIV, (ii) pregnant women first diagnosed with HIV during their current pregnancy, (iii) pregnant women first diagnosed with HIV during their current pregnancy and deciding to continue their pregnancy and deliver the baby, and (iv) women already known to have HIV who get pregnant and deliver a baby.

Surveillance in Germany provides data on newly diagnosed HIV infections among children and newborns and data on newly diagnosed HIV infections among women. However, surveillance data neither provide information on the pregnancy status of women diagnosed with HIV nor complete information on the number of newborns potentially exposed to HIV during pregnancy. Thus, while calculating the second of the two WHO indicators (HIV MTCT case rate) has been possible, the denominator for the first indicator (MTCT rate) has been missing.

Healthcare reimbursement data are one data source that might help fill this gap. Health insurance, either through statutory health insurance (SHI) or private health insurance, is obligatory in Germany. SHIs cover ca 85–90% of pregnancies in Germany while private health insurances cover ca 10%. The vast majority of the population is therefore covered by health insurance. We aimed to use healthcare reimbursement data from a large sample of women who received pregnancy-related healthcare to estimate: (i) the proportion of all pregnancies and pregnancies resulting in child birth screened for HIV, (ii) the number of newly diagnosed HIV infections among pregnant women, and (iii) the number of women with an already-diagnosed HIV infection becoming pregnant across a period of 5 years, from 2011 through 2015.

## Methods

In our retrospective observational study, we used data from national population statistics, healthcare reimbursement data from a large sample of women who received pregnancy-related healthcare, transmission group-specific HIV prevalence estimates for women of child-bearing age (i.e. 15–49 years) from the early 1990s to the present from the German HIV epidemic model, which is a modified back-calculation model, and HIV reporting data of perinatally infected children from the anonymous HIV registry at the Robert Koch Institute.

### Data sources and calculations

#### Total number of pregnancies and pregnancy outcomes in Germany

The total number of pregnancies and pregnancy outcomes for 2001 through 2016 were obtained from official German population statistics [11]. Number of births was transformed to number of pregnancies by adjusting for twin and triplet births.

#### Number of pregnant women with HIV

SHI reimbursement data from a large sample of women who received pregnancy-related healthcare with HIV-specific reimbursement codes were used to estimate the number of already known and newly discovered HIV infections among pregnant women for 2011 to 2015.

For 1993 to 1998, a research project in the federal states of Berlin and Lower Saxony screened all residual blood samples from phenylketonuria (PKU) screening for HIV antibodies [12]. From these data, HIV prevalence among women giving birth was determined for these years and federal states. However, whether the women were aware of having HIV is not known for these data. No information on the number of premature pregnancy terminations among women living with HIV in these two federal states during this period was available.

#### Number of HIV screening tests during pregnancies reimbursed by statutory health insurances and by private health insurance

The total number of HIV screening tests during pregnancies reimbursed by SHI in Germany was queried from Kassenärztliche Bundesvereinigung (KBV), a central agency that collects all SHI reimbursement claims, for 2001 through 2016. However, the actual timing of the HIV screening test is not reported and may be delayed by late presentation of pregnant women for antenatal care.

The denominator for the HIV screening tests is unclear for these data and is somewhere between the total number of pregnancies among SHI-covered women and the total number of pregnancies resulting in live births, but probably closer to the number of pregnancies. So to estimate the denominator, the number of pregnancies covered by SHI, two methods were used: (i) subtraction of the estimated number of newborns covered by private health insurance (see below) from the total number of live births as obtained from official population statistics and (ii) the number of syphilis screening tests reimbursed by SHI as reference, assuming that syphilis screening in pregnancy has reached an almost universal coverage.

As there are no data on the number of pregnancies covered by private health insurance, the number of newborns covered by private health insurance was used as a proxy. The latest available estimate was for the years 2000 through 2010 [13]. For the estimate from 2001 to 2016, it was assumed that the proportion of women giving birth and covered by private health insurance remained unchanged from the 2010 levels. There are also no available data on HIV screening during pregnancy for privately ensured women. Since private health insurance reimbursements are generally higher than SHI reimbursements, there is a financial incentive for healthcare providers to offer at least the same services for privately ensured clients. For our analysis, it was assumed that the HIV screening rate among privately insured women is equivalent to that among SHI-covered women.

Some pregnant women with assumedly increased risk of HIV MTCT, such as asylum seekers, undocumented women and women from European Union (EU) countries without valid health insurance in Germany are not included in either of these two datasets.

#### Pregnancies resulting in live births with HIV screening tests performed

About 87% of German inhabitants (70.3 million) are insured at one of 123 German SHIs. To estimate the proportion of pregnancies resulting in live births in which an HIV screening test was ordered, health insurance reimbursement data for a subsample available in the database of the German Health Risk Institute (HRI) were analysed. As at 31 December 2013, 82 German SHIs contribute longitudinal data from ca 6 million Germans. The HRI database provided data on 6.9% of the pregnancies that resulted in live births in Germany in the period 2011 to 2015.

Data are anonymised with respect to individual insurant, healthcare provider (e.g. physicians, practices, hospitals and pharmacies) and the respective SHI. In addition to anonymisation, access to the data is strictly controlled. It is prohibited to transmit patient-level data, and all data analyses had to be performed by HRI employees or trained associated researchers.

The estimated delay of data being available in the database is ca 3 to 9 months, e.g. healthcare data until 31 December 2015 can be expected to be available in the database at September of 2016 at the latest.

The following was extracted from the HRI database: (i) demographic information, i.e. sex, age, region of residence, of all insured individuals alive on 31 December 2013 and (ii) outpatient services and diagnoses as well as hospital case information including admission and discharge dates, major and minor diagnoses, and diagnostic and therapeutic services performed between 2010 and 2015. The ICD codes used to extract information on pregnancies and HIV from the HRI database are listed in Table 2.

**Table 2:** EBM codes and ICD-10 diagnoses used for retrieval of data on pregnancies and HIV from the Health Risk Institute, Germany, 2011–2015

Feature	Catalogue	EBM code/ICD-10 diagnosis	Designation
Screening	EBM	01770	Care for a pregnant woman
	EBM	32007	Check-ups in accordance with the German maternity guidelines
Birth	ICD-10	O80	Spontaneous delivery of a singleton
	ICD-10	O81	Birth of a singleton by forceps or vacuum extraction
	ICD-10	O82	Birth of a singleton by Caesarean section
	ICD-10	Z37.0/2/3/5/6/9	Live-born singleton/twin/multiple
	ICD-10	Z38^a^	Live births by birthplace
HIV test	EBM	01811	HIV immunoassay
	EBM	32575	HIV-1 or HIV-1/2 antibody immunoassay
	EBM	32576	HIV-2 antibody immunoassay
	EBM	32783	Detection of HIV
HIV confirmatory test	EBM	32824	HIV RNA
	EBM	32660	HIV-1, HIV-2 antibody Western blot
Exclusion criteria			
HIV-specific services	EBM	30920	Flat rate for the treatment of HIV-infected persons
	EBM	30922	Supplement I^b^ for the treatment of HIV-infected persons
	EBM	30924	Supplement II^b^ for the treatment of HIV-infected persons
Pre-existing HIV diagnosis	ICD-10	O98.7	HIV disease complicating pregnancy, childbirth and puerperium
	ICD-10	R75	Laboratory evidence of HIV
	ICD-10	B20-B24	Infectious and parasitic diseases/malignant neoplasms/other diseases because of HIV disease
	ICD-10	Z21	Asymptomatic HIV infection
	ICD-10	U60	Clinical categories of HIV disease
	ICD-10	U61	Number of CD4^+^ T cells in HIV disease

EBM: Einheitlicher Bewertungsmaßstab (statutory insurance fees); ICD: international classification of diseases.

^a^ The ICD-10 code Z38 should generally be used to encode childbirth in the newborn. Since newborns often do not have their own insurance number at the beginning of life, billing is carried out via the maternal insurance number. To be certain to capture all births, this ICD-10 code was also included in the case definition.

^b^ The supplements are bonus payments to cover the increased demand of time and other resources for the care of HIV-infected patients.

A statistical approach was used to extrapolate the data generated from the HRI database subsample to the general population or to all pregnancies by weighting the data: results were standardised for age. Weights were calculated for the insured population of the HRI database by adjusting to the age and sex distribution of the total population in Germany from 2011 to 2015.

#### HIV-diagnosed and HIV-infected women

Based on a modified back-calculation model, the Robert Koch Institute (RKI) has been estimating the course and status of the HIV epidemic in Germany since 2010. For this analysis, data reported as at the end of 2016 was used to estimate the prevalence of HIV diagnoses and infections in women aged 15 to 49 years by federal state, transmission risk and region of origin [14].

#### Women living with HIV and giving live birth

The proportion of women living with HIV and giving live birth (numerator: number of women living with HIV giving live birth; denominator: estimated number of women in the age group 15 to 49 years of age living with HIV in Germany) was estimated. For 1993 to 1998, this was based on data from the anonymous unlinked HIV testing of left-over samples from PKU screening of newborns and the prevalence rates of women living with HIV from the RKI HIV estimate for the two federal states of Berlin and Lower Saxony [12]. For 2011 to 2015, the estimated number of HIV pregnancies from the health insurance reimbursement data and the estimated total number of women living with HIV in the age group 15 to 49 years available from the RKI epidemic model were used. For both periods, a mean value was determined. The evolution of the calculated proportion in the years between (1999–2010) and after (2016) those two periods was imputed considering the composition of women of childbearing potential by region of origin and risk of infection.

#### HIV diagnoses among children born in Germany

Data on HIV diagnoses among children born in Germany between 2001 and 2016 were taken from the anonymous, statutory HIV infection registry at the RKI. Since 2004, infections reported from children younger than 15 years of age have been verified by contacting the reporting physician regarding age, place of birth, and any information available on possible reasons for late diagnosis or failure of established procedures to prevent MTCT of HIV.

#### HIV mother-to-child transmission rates

HIV MTCT rates for 2001 to 2016 were estimated by dividing the number of HIV infections diagnosed among children born in a given year in Germany and the estimated number of women living with HIV and giving live birth in that year.

#### Availability of additional data and material

Additional data tables including birth statistics, healthcare reimbursement data, data on a HIV pregnancy cohort from the SHI- subsample, estimates on the number of women living with HIV in Germany from mathematical modelling, measured and imputed fertility rates for women living with HIV in Germany, and data on HIV infections in children born in Germany and reported to the HIV registry at the RKI are available as Supplementary material.

### Ethical statement

PKU samples tested in the study from 1993 to 1998 were anonymised and unlinked before HIV testing. Other data used are either healthcare reimbursement data or estimates based on surveillance data. Hence, no ethics approval or individual consent was required.

## Results

According to German national population statistics, the number of pregnancies in Germany from 2001 to 2016 fluctuated between 768,464 in 2011 (minimum) and 887,115 in 2016 (maximum). The number of pregnancies resulting in live birth ranged between 657,210 in 2011 and 785,480 in 2016.

### Proportion of pregnancies with HIV screening, 2001–2016

**Figure 1:**
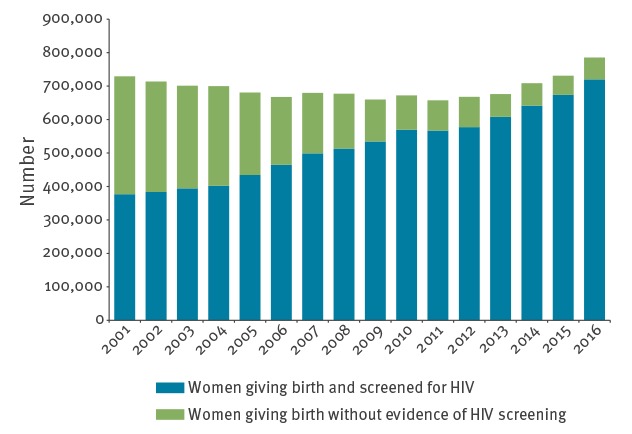
Number of pregnancies resulting in live birth and estimated proportion screened for HIV, Germany, 2001–2016 (n = 11,107,882)

The estimated proportions of women giving live birth who were screened for HIV during 2001 to 2016 is shown in Figure 1. The number of HIV screening tests reimbursed by SHI increased from 398,784 in 2001 to 711,826 in 2016. Assuming an equal screening rate for women covered by private health insurance, the proportion of women giving live birth and screened for HIV during pregnancy increased from 52% (376,660/729,171) in 2001 to 92% (720,313/785,480) in 2016.

**Figure 2:**
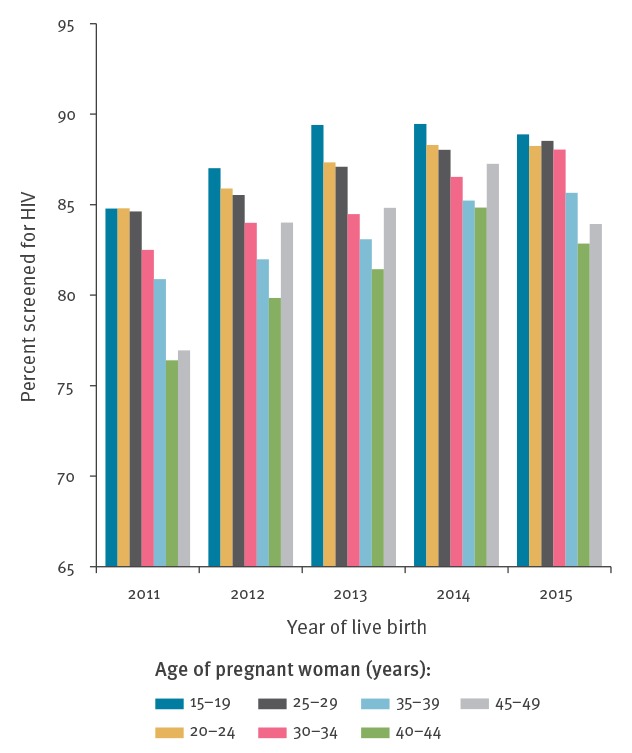
HIV testing rates in women giving live birth, by age group, statutory health insurance subsample, Germany, 2011–2015 (n = 203,160)

The proportion of pregnant women giving live birth that had been screened for HIV in our SHI sample increased slowly but consistently from 82.3% in 2011 to 87.0% in 2015 (Supplementary Table S1). Extrapolated to all live births, this would still leave between 114,000 and 89,000 pregnant women giving birth unscreened. Testing rates differed by age group, with lower rates in older age groups (Figure 2).

The statutory health insurance subsample covers 6.9% of all pregnancies in Germany during 2011–2015.

### Pregnant women with HIV in the statutory health insurance subsample, 2011–2015

The proportion of pregnant women giving live birth with a reactive HIV screening test result requiring confirmatory testing ranged between 0.09% (n = 492) and 0.14% (n = 725), with no clear time trend (Supplementary Table S1). During the 5-year period, the mean proportion of confirmatory tests that resulted in HIV diagnosis in health insurance data was 19% (range: 11–26%). This corresponds to HIV diagnosis rates between 0.02% and 0.07% of the women tested (Supplementary Table S1).

The mean estimated yearly number of new HIV diagnoses among pregnant women in Germany between 2011 and 2015 was 130, ranging between 68 and 201. In the same period, the estimated mean number of women with already known HIV diagnosis who became pregnant and gave live birth was 354 per year, with a range between 276 and 434. A mean number of 484 pregnancies among women living or newly diagnosed with HIV was observed per year in the period 2011 to 2015. The majority, 73% (1,771/2,420), were pregnancies in women already diagnosed with HIV while 27% (648/2,420) were detected by pregnancy screening (Supplementary Table S1).

### Number and composition of HIV-diagnosed and HIV-infected women, 1993–2016

**Figure 3:**
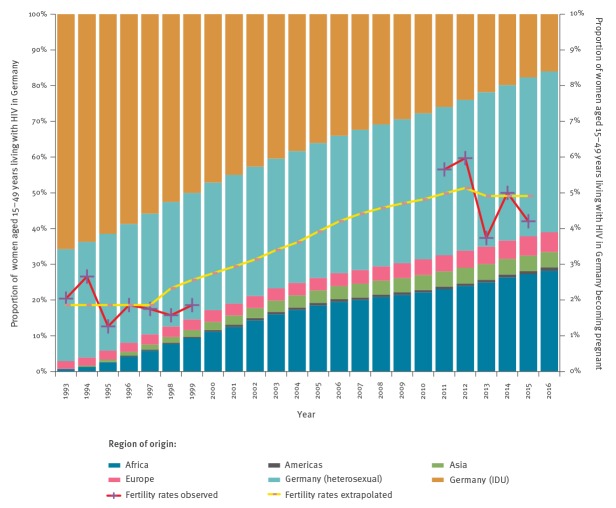
Composition of the 15–49 year age group of women living with HIV, by transmission risk factor and region of origin, and estimated proportion becoming pregnant per year, Germany, 1993–2016

The estimated number of women in the age group 15 to 49 years living with HIV in Germany increased from ca 6,000 in 1993 to almost 11,000 in 2016 (Supplementary Table S2). The composition of these women changed considerably over time; the proportion of women who became infected while injecting drugs intravenously decreased from 65% (n = 3,880) in 1993 to 16% (n = 1,730) in 2016 while the proportion of women who acquired HIV sexually within Germany, Africa, Asia or other European countries increased accordingly (Figure 3, Supplementary Table S2).

IDU: injecting drug use.

Fertility rate is defined as the number of pregnancies/number of women aged 15–49 living with HIV per year.

The mean proportion of women living with HIV who gave live birth in the period 1993 to 1998 in the federal states of Berlin and Lower Saxony was 1.9% (range: 1.6–2.7%) based on the estimated number of women living with HIV in the two states and the number of HIV-positive test results in the left-over samples from the PKU newborn screening study in these years (Figure 3). The estimated proportion of women living with HIV giving live birth in the period 2011 to 2015 was close to 5% (range: 3.7–6.0%) based on health insurance data and the prevalence in the epidemic model. It can be concluded that the proportion of women giving live birth among women living with HIV in Germany increased considerably between 2000 and 2010, and the evolution of this proportion was estimated based on an assumed linkage with the proportion of women who acquired HIV sexually. During the time period 1999 to 2015, the proportion of women giving live birth among all women aged 15–45 years ranged between 4% and 5%.

In an estimated 13.1% of the pregnancies with a reactive HIV screening test of the mother in the 2011 to 2015 health insurance subsample, the pregnancy was prematurely terminated.

### HIV mother-to-child transmission rates, 2001–2016

The number of children born in Germany between 1999 and 2016 who had acquired HIV perinatally and were diagnosed and reported by 31 December 2018 was 164. The HIV case rates (number of HIV MTCT per 100,000 live births) for 2001 to 2016 are shown in Table 3.

**Table 3:** HIV case rate, HIV mother-to-child transmission per 100,000 live births, Germany, 2001–2016

**Year**	**Number of live births**	**HIV MTCT cases**	**HIV MTCT per 100,000 live births**
2001	734,475	15	2.042
2002	719,250	14	1.946
2003	706,721	12	1.698
2004	705,622	16	2.268
2005	685,795	13	1.896
2006	672,724	16	2.378
2007	684,862	6	1.314
2008	682,514	5	1.172
2009	665,142	8	1.353
2010	677,947	6	1.033
2011	662,685	5	0.905
2012	673,544	8	1.188
2013	682,069	2	0.293
2014	714,966	5	0.699
2015	737,575	3	0.407
2016	792,495	6	0.757

MTCT: mother-to-child transmission.

**Figure 4:**
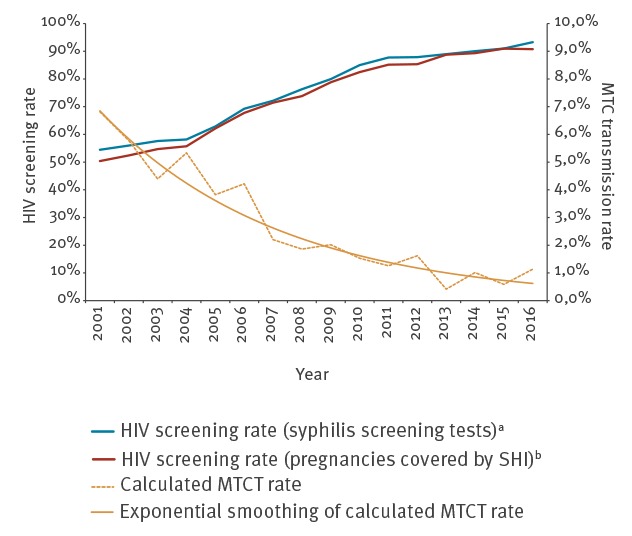
Estimated HIV screening rates in pregnancy and HIV mother-to-child transmission rates, Germany, 2001–2016

MTCT: mother-to-child transmission; SHI: statutory health insurance.

^a^ Numerator: HIV screening tests reimbursed by SHI; denominator: number of syphilis screening tests reimbursed by SHI. The latter was used, based on the assumption of almost universal coverage of syphilis screening in pregnancy.

^b^ Numerator: HIV screening tests reimbursed by SHI; denominator: estimated number of pregnancies covered by SHI.

Based on the estimated number of women living with HIV in Germany, becoming pregnant and giving live birth (estimated proportion of women living with HIV and giving live birth × number of women living with HIV in Germany and aged 15–49 years) the MTCT rates for 2001 through 2016 were calculated. The rates dropped from 6.8% in 2001 to values fluctuating between 0.4% and 1.1% in 2013 to 2016 (Figure 4, Supplementary Table S2). In the same period, the HIV screening rates in pregnancy had increased from ca 50% to ca 90% (Figure 4).

In the period 2011 to 2015, 24 children were born with HIV in Germany. In 12 cases, the mother had been newly diagnosed with HIV during pregnancy, in five cases, before she became pregnant. This represents an overall estimated HIV transmission rate of 1.0%, with 0.3% among women already diagnosed with HIV, and 1.9% among women diagnosed during pregnancy (Supplementary Table S1). For the remaining seven cases, the HIV status of the mother was unknown at the time of birth.

In recent years, only five of 24 HIV MTCT cases could be attributed to lack of HIV screening, while 19 cases were because of various other reasons (Supplementary Table S1). The single most important of these other reasons was difficulty to access prenatal care in time because of migration-related access barriers.

## Discussion

The coverage of pregnancies with HIV screening tests has improved considerably over the study period 2001 to 2016, but a sizeable proportion of ca 10% of deliveries in 2016 still appeared to occur without evidence of HIV testing of the mother. Assuming that failure to test for HIV in these 10% is at random, the number of missed maternal HIV infections would have been around 37 in 2011 and 2012, declining to 10–15 in the years 2013 to 2016. This could have resulted in ca 3–5 MCTC annually in the years since 2013. According to the experience of the years since 2011, the number of infections in children because of lack of screening of the mothers was lower: the maximum number observed per year was two, although it cannot be excluded that some infections have not yet been diagnosed. It appears more likely that in most cases when HIV tests are not offered or not accepted, healthcare providers and/or pregnant women rightly assume that their odds of having HIV are very low. However, at least one to two HIV MTCT per year could be avoided if the remaining 10% of pregnancies would be covered with HIV screening.

The discrepancies between the different methods to estimate the coverage of pregnancies with HIV screening are mainly because of uncertainties about the exact proportion of pregnancies that were covered by SHI in the period 2011 to 2016. It was assumed that the proportion remained constant at the 2010 level, but since 2011, Germany has experienced increased immigration and it is likely that recent immigrants would be disproportionally covered by SHI or other mechanisms, e.g. publicly funded healthcare for asylum seekers.

The number of confirmatory tests for HIV among pregnant women was approximately five- to six fold higher than the number of new HIV diagnoses. There may be different explanations for this discrepancy: repeat confirmatory testing, e.g. because of referral to an HIV specialist after primary diagnosis or, probably more relevant, the low positive predictive value of reactive HIV screening tests because of the low prevalence in the population screened.

Approximately one in three pregnancies of women living with HIV is newly diagnosed during that pregnancy. However, the proportion of women being diagnosed with HIV during pregnancy and not carrying on their pregnancy in the period 2011 to 2015 did not differ from the proportion of women not diagnosed with HIV (13% vs 12–15%).

Our data suggest a more than twofold increase of fertility rates among the population of women living with HIV in Germany from the end of the 1990s until 2015. In the author’s opinion, this is probably because of at least two factors. One is the changing composition of the female population living with HIV. In the 1980s and 1990s women acquired HIV predominantly by IDU, whereas now increasing proportions of women acquire HIV sexually. The second factor could be that women who inject drugs more often terminate pregnancies prematurely [15,16] and migration to Germany from regions with higher mean fertility rates than in Germany [17,18] and high HIV prevalence in the general population has increased [19,20]. Similar changes in the female population living with HIV have been reported from other western European countries [21]. Among women living with HIV, the mean proportion giving live birth per year between 2011 and 2015 was 4.9%, which is very close to the proportion calculated for women from the general population in Germany for this period [22]. Since it was not possible to control the German fertility rates for region of origin of the mother, it remains unclear whether and to what extent fertility rates of women living with HIV in Germany are lower than their uninfected peers. Comparative analysis of fertility rates of women living with HIV and other women from countries in sub-Saharan Africa, the Caribbean, and South-East Asia based on data collected in Demographic and Health Surveys (DHS) concluded that fertility rates of women living with HIV in these countries were generally lower than their uninfected peers [23]. However, most of these DHS were conducted more than a decade ago, and the reasons for lower fertility with HIV diagnosis include behavioural changes after HIV diagnosis. Improved quality of life, increased life expectancy and improved prospects to avoid HIV MTCT with improved HIV treatment, may impact on the desire of women living with HIV to have children [4,24].

Although the female child-bearing population living with HIV has almost doubled and the proportion of women giving live birth in this population has almost tripled, the number of HIV-infected children, the case rate of HIV MTCT and the rate of HIV MTCT have decreased to levels below the targets foreseen by the WHO for the European Region. This is most likely because of the increasing HIV screening rates during pregnancy and the highly effective medical interventions such as antiretroviral treatment of pregnant women to prevent HIV MTCT.

### Limitations

The number of children who acquired HIV perinatally in Germany may be underestimated, particularly for more recent years, because some infections are still undiagnosed. To minimise this problem, the analysis was restricted to children born before 2017 and included children reported until end of 2018, thus allowing for a diagnosis delay of up to 2 years.

The extrapolated numbers of women being diagnosed with HIV during pregnancy and those living with diagnosed HIV and becoming pregnant, based on the SHI subsample from 2011 to 2015 may be underestimated because of a biased distribution in different SHIs. While the number of pregnancies in our SHI subsample remained stable across the 5-year observation period, population statistics demonstrated an increase in the number of pregnancies. This increase is at least partly associated with increased immigration [18,25], and women who have recently immigrated may be less represented in the SHIs constituting our SHI subsample. In addition, women living with HIV and becoming pregnant may not yet be covered by SHI but by publicly funded healthcare for asylum seekers, particularly in the years 2014 to 2016. Other groups not covered by SHI in Germany and thus lacking access to healthcare are undocumented migrants and EU citizens without valid health insurance.

## Conclusions

The expansion of HIV screening in pregnancy in combination with highly effective medical interventions to prevent HIV MTCT has successfully minimised HIV infections among newborns. To achieve further progress towards elimination of MTCT of HIV in Germany, all pregnant women need to be offered HIV screening and healthcare access barriers for migrant women and asylum seekers need to be reduced.

This analysis is contingent upon the particularities of the German healthcare system. While improved HIV screening of pregnant women and general trends such as the shift in HIV transmissions from drug use-associated risks to sexually acquired infections in women may be generalisable to other EU countries, efforts to eliminate HIV MTCT in other healthcare systems may differ. Compiling an overview of HIV MTCT elimination efforts across all EU countries was beyond the scope of this analysis, but it would be desirable to have such an overview at the European level.

## Supplementary Data

620000 Supplementary Material
