# Supplementary material for: HIV infections and HIV testing during pregnancy, Germany, 1993 to 2016
Source: Euro Surveill. 2019 Nov 28;24(48):1900078. doi: 10.2807/1560-7917.ES.2019.24.48.1900078 (PMC6891947; doi:10.2807/1560-7917.ES.2019.24.48.1900078)
Supplement: Supplementary Material [file 1900078_MARCUS_HIV_SupplementaryMaterial.pdf]

Supplementary Material

This supplementary material is hosted by Eurosurveillance as supporting information alongside the article ‘HIV infections and HIV testing among pregnant women and during pregnancies in Germany – progress and challenges’ on behalf of the authors who remain responsible for the accuracy and appropriateness of the content. The same standards for ethics, copyright, attributions and permissions as for the article apply. Supplements are not edited by Eurosurveillance and the journal is not responsible for the maintenance of any links or email addresses provided therein.

Table S1: Data on pregnancies, HIV screening, life births, and HIV infections transmitted to new-borns from national population statistics, a health insurance sample, and the HIV case registry for the period 2011-2015

	2011	2012	2013	2014	2015	total across 5 years	mean per year	proportions
**National population/health care statistics**								
pregnant women	768,464	777,087	781,519	810,976	833,085	3,971,131	794,226	n.a.
women with life births	657,210	667,872	676,161	708,664	731,061	3,440,968	688,194	n.a.
HIV screening tests in pregnancy reimbursed by SHI	580,115	587,735	615,093	642,428	671,975	3,097,346	619,469	n.a.
proportion of pregnancies screened*	83.9%	84.0%	87.4%	88.0%	89.6%	n.a.	88.6%	n.a.
**Health insurance sample**								
pregnancies	56,625	56,901	56,539	56,710	55,201	281,976	56,395	n.a.
life births	48,156	48,346	48,039	48,217	46,964	239,722	47,944	7.0%
HIV screening tests in pregnancy reimbursed	44,000	44,801	45,183	46,220	45,427	225,631	45,126	80.0%
HIV screening tests in pregnancy reimbursed in women giving birth	39,635	40,361	40,670	41,618	40,876	203,160	40,632	84.7%
HIV screening tests extrapolated)	566,050	583,835	588,630	616,437	630,791	2,985,743	597,149	n.a.
95% CI	560,802 – 571,300	578,488 – 589,209	583,236 – 594,013	610,863 – 622,031	625,026 – 636,552	n.a.	n.a.	n.a.
extrapolated number of life births	616,725	627,636	624,260	641,579	650,890	3,161,090	632,218	91.9%
extrapolated number of HIV screening tests in women giving birth	512,387	528,983	532,859	557,833	570,493	2,702,555	540511	78.5%
95% CI	507,378 – 517,373	523,860 – 534,083	527,732 – 538,005	552,533 – 563,173	565,013 – 575,997	n.a.	n.a.	n.a.
proportion of life births screened extrapolated	82.3%	83.5%	84.7%	86.3%	87.0%	n.a.	84.8%	n.a.
estimated number of life births going unscreened	104,338	98,653	91,401	83,746	80,397	458,535	91,707	14.5%

**Health insurance sample: HIV infections newly or previously diagnosed in women**								
extrapolated number of pregnant women with confirmatory HIV test	717	826	653	647	668	3511	702	12.8%
95% CI	535 - 911	608 - 1009	477 - 839	501 - 851	495 - 876	n.a.	n.a.	n.a.
extrapolated number of women with life births and confirmatory HIV test	646	725	585	492	614	3,062	612	n.a.
95% CI	443 - 791	568 - 939	394 - 729	362 - 665	409 - 762	n.a.	n.a.	n.a.
Proportion of HIV screening tests requiring confirmation testing	0.13%	0.14%	0.11%	0.09%	0.11%	0.11%	0.11%	n.a.
women newly diagnosed with HIV during pregnancy in health insurance sample	13	13	7	5	8	46	9	n.a.
extrapolated number of new HIV diagnoses among all pregnant women	183	201	89	68	107	648	130	n.a.
95% CI	*133 - 301*	135 - 306	13 - 133	13 - 137	13 - 140	n.a.	n.a.	n.a.
already diagnosed HIV infections in health insurance sample	27	29	23	30	28	137	27	n.a.
extrapolated number of already diagnosed HIV infections among all pregnant women	358	373	276	434	330	1771	354	73.2%
95% CI	228 - 495	233 - 503	172 - 413	313 - 589	163 - 447	n.a.	n.a.	n.a.
extrapolated total number of HIV infections among pregnant women	541	574	365	502	437	2419	484	n.a.
Proportion of HIV pregnancies newly diagnosed in current pregnancy	33.8%	35.0%	24.4%	13.5%	24.5%	26.8%	26.8%	n.a.
HIV infections newly diagnosed per screened pregnancies	0.04%	0.04%	0.02%	0.01%	0.02%	0.02%	0.02%	n.a.
upper limit of estimated maternal HIV infections potentially missed due to lack of screening	37	37	15	10	15	115	23	n.a.
**infections transmitted to new-borns**								

newly diagnosed HIV transmissions to children born in Germany (in the respective year)	6	8	2	5	3	24	5	1.0%
maternal infection newly diagnosed in pregnancy	2	4	1	2	2	12	2	1.9%
maternal infection already known	2	1	0	2	0	5	1	0.3%
due to not offering an HIV test	1	2	1	1	0	5	1	5/24
Migration/ health care access-related	2	1	0	3	2	8	2	8/24

n.a.= not applicable; 95% CI = 95% Confidence Interval

* (SHI, assumed 90% population coverage, assumed one test/pregnancy)

Supplementary Table S2: Data on number and composition by transmission risk of women of child-bearing age (15-49 years) from the RKI epidemic model for HIV in Germany, data on measured and imputed fertility rates among women living with HIV, and data on deliveries and infections transmitted to new-borns among women living with HIV in Germany between 1993 and 2016

Year	estimated number of women living with HIV in Germany (1)	estimated number of female IDU living with HIV (1)	estimated number of women with sexually acquired HIV (1)	Proportion of women living with HIV and giving birth data-based (2)	Proportion of women living with HIV and giving birth imputed	data-based number of deliveries from HIV pregnancies (2)	imputed number of deliveries from HIV pregnancies	Number of new-borns acquiring HIV in Germany (3)	HIV MTCT rate
1993	5895	3880	2015	2.04%	1.86%	120	118	n.a.	n.a.
1994	6050	3835	2215	2.66%	1.86%	161	121	n.a.	n.a.
1995	6115	3765	2350	1.26%	1.86%	77	122	n.a.	n.a.
1996	6230	3675	2555	1.86%	1.86%	116	125	n.a.	n.a.
1997	6485	3625	2860	1.75%	1.86%	114	130	n.a.	n.a.
1998	6790	3565	3225	1.57%	2.33%	107	136	n.a.	n.a.
1999	7085	3530	3555	n.a.	2.56%	n.a.	181	12	6.62%
2000	7285	3455	3830	n.a.	2.75%	n.a.	200	3*	1,50%*
2001	7490	3380	4110	n.a.	2.94%	n.a.	220	15	6.81%
2002	7730	3335	4395	n.a.	3.13%	n.a.	242	14	5.78%
2003	8035	3255	4780	n.a.	3.40%	n.a.	273	12	4.39%
2004	8300	3205	5095	n.a.	3.61%	n.a.	300	16	5.33%
2005	8680	3145	5535	n.a.	3.92%	n.a.	340	13	3.82%
2006	9025	3070	5955	n.a.	4.20%	n.a.	379	16	4.22%
2007	9255	2995	6260	n.a.	4.41%	n.a.	408	6	1.47%
2008	9400	2905	6495	n.a.	4.57%	n.a.	430	5	1.16%
2009	9480	2800	6680	n.a.	4.70%	n.a.	446	8	1.79%
2010	9495	2650	6845	n.a.	4.81%	n.a.	457	6	1.31%
2011	9570	2480	7090	5.65%	4.98%	541	477	6	1.26%
2012	9615	2305	7310	5.97%	5.13%	574	494	8	1.62%
2013	9775	2135	7640	3.73%	4.91%	365	480	2	0.42%
2014	10045	1985	8060	5.00%	4.91%	502	493	5	1.01%
2015	10400	1850	8550	4.20%	4.91%	437	511	3	0.59%
2016	10775	1730	9045	4.49%	4.91%	484	529	6	1.13%

n.a.= not available

(1) data source: HIV epidemic model, RKI 2017

(2) HIV screening of left-over samples from PKU new-born screening Berlin/Lower Saxony (1993-1998); SHI subsample (2011-2015)

(3) anonymous HIV case registry, RKI (31.12.2017)

* reporting data not reliable for this year due to change in notification system
